# Treatment of Second to Third-Degree Burns in A 2-Day-Old Infant: A Case Report

**DOI:** 10.29252/wjps.9.1.82

**Published:** 2020-01

**Authors:** Thomas Ziegler, Thomas Cakl, Johannes Schauer, Dieter Pögl, Ahmad Abdelkarim, Tomas Kempny

**Affiliations:** Division of Plastic and Reconstructive Surgery, Department of Surgery, Klinikum Wels-Grieskirchen, Austria

**Keywords:** Burn; Newborn, Neonatal, Infant, Wound, Repair

## Abstract

Burn injuries in newborns are particularly complex cases. Since these patients are rare, there is little experience and no existing standardized treatment. This report examines a case of accidental second to third-degree burning of the heel and toes on the left foot in a new-born girl. The burns covered an estimated 1% of the total body surface area (TBSA). After an initial debridement and 32 days of non-surgical wound therapy with Adaptic® fat gauze dressings, we were able to achieve an aesthetically and functionally satisfactory result including the complete preservation of all toes. Modern wound treatment following the principle of less frequent dressing changes allows the burn wound to have better re-epithelialization. New findings in stem cell research indicate that the high proportion of mesenchymal stem cells (MSC) in postnatal blood is also involved in the regeneration and healing of burns. To our knowledge, this is the first case report dealing with initial non-surgical combustion therapy in a newborn. In order to eliminate a scar contracture, we carried out a Z-plasty one year later.

## INTRODUCTION

Burns in children can have far more serious consequences than in adults, since even small burns already occupy a large percentage of the body surface.^1^ Depending on exposure time and intensity, the thermal damage can affect purely the superficial papillary as well as the deep reticular dermis. The healing of such wounds usually takes 2 to 3 weeks. In superficial burns, no marked scarring is to be expected. In most cases high-grade burns affect relatively small percentages of the skin surface.^2^^,^^3^

In 2013, the rate of burn mortality in children aged 1 to14 years was 2.5 per 100.000 worldwide, whereas in the high-income Organisation for Economic Co-operation and Development (OECD) area, it was only 0.4.^4^ The most common burned region in children is the trunk (23.4%), followed by the hand (18.8%). The child‘s foot is ranked 6^th^ (7.7%). Children aged 0 to 3 years is the group (69.4%) who is most likely to suffer burns.^2^ Burns in newborns, however, are rare and occur mainly in hospital setting.^5^ The local standard therapy for high-grade burns includes immediate debridement and coverage with suitable dressings.^3^

The task of wound dressings is to prevent transdermal fluid loss and infections and to enable re-epithelialization as well as possible pain-free dressing changes. Simple application and cost-effectiveness are also important factors.^3^ We report our experience with a newborn child who was treated by us because of an iatrogenic second to third-degree burn on the left foot.

## CASE REPORT

The patient was released by caesarian section and the postnatal adjustment went without problems. Warming the heel for capillary blood gas control on the 2^nd^ postnatal day with a heat pad caused severe burns on the left foot. The blisters were removed at the Department of Pediatrics and treated with Jelonet^TM^ (Smith & Nephew plc, London, UK) a sterile Paraffin-impregnated gauze. On the 4^th^ postnatal day, our division was consulted. We found superficial partial, deep partial and full thickness burning areas on the plantar forefoot, the toes, the lateral foot edge and on the heel. 

The extent of the burn was about 1% of the body surface. Since the debridement was already performed, we started a conservative treatment with Adaptic® (Systagenix Wound Management Limited, Gatwick, UK)- a small mesh sized non-adhering dressing made of cellulose acetate fabric and impregnated with petrolatum emulsion. Two days later, the wound had deteriorated. Dry necroses occurred in the burnt areas. The tip of the big toe and the 3^rd^ toe, the lateral half of the small toe, the lateral foot margin and a region of 2×2 cm at the heel were necrotic. 

**Fig. 1:**
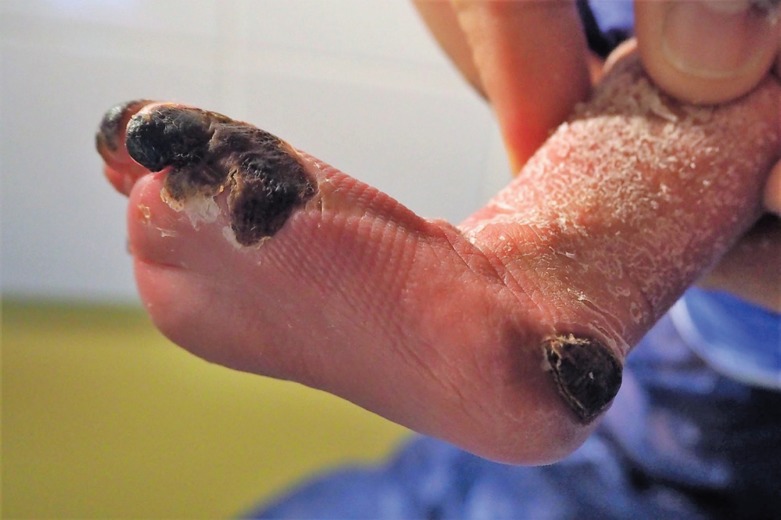
Left foot of a newborn child after second degree burn of the big toe, tip of the third, fourth and fifth toe, lateral foot margin and the heel on the sixth day of treatment with Adaptic®.

**Fig. 2:**
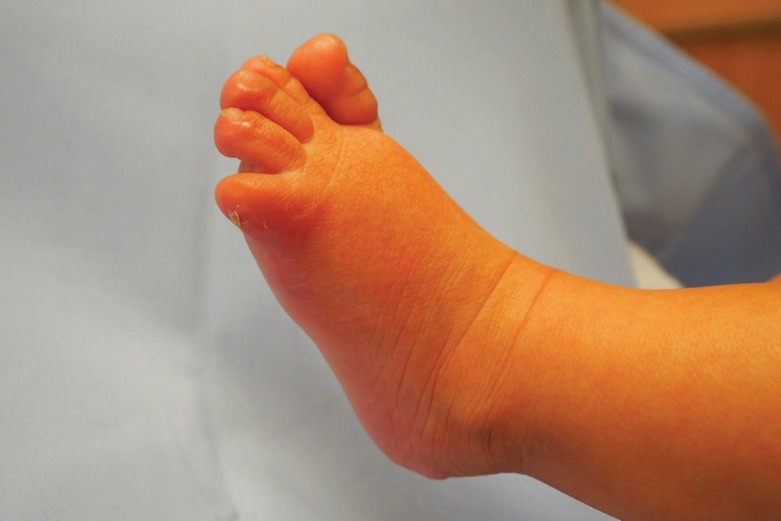
Lateral foot margin three months after burn. Lateralization of the toenail and complete re-epithelialization

**Fig. 3:**
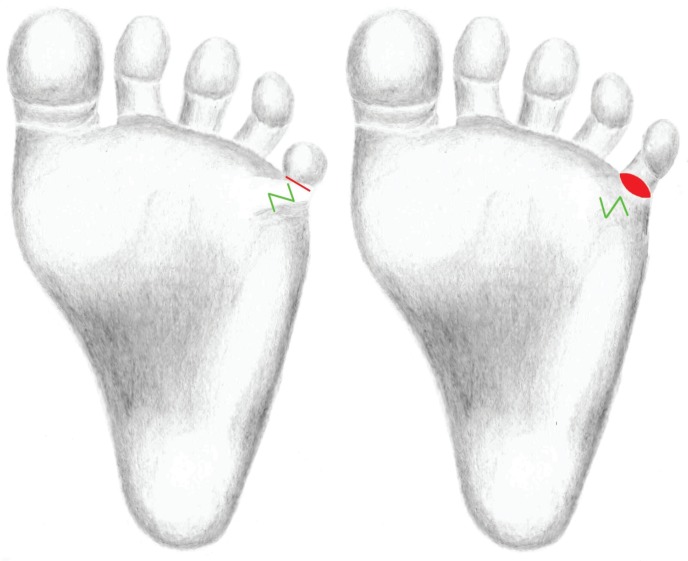
Surgical planning of the Z-plasty along the post-burn scar contracture and the marking of the incision along the shortened flexion fold. After performance of the Z-plasty to release the post-burn scar contracture. The full thickness skin graft was inserted into the incision defect after stretching the small toe in full extension

The extent of the necrosis in the depths of the small toe could not be estimated at this point. (Figure 1-3). As a radical debridement would possibly have extended to the base joints of the toes and the heel bone, the necrosis plates were left in place. In addition, antibiotic prophylaxis with cefuroxime was initiated at the 7^th^ postnatal day. The parents were informed about the baby’s possible loss of the big and small toe. Dressing changes with Adaptic® were carried out in 48-hour intervals. To minimize scar contracture, the small toe was carefully placed in neutral-position during dressing changes and underlaid with Adaptic® at the flexion fold. 

Thanks to the resulting improvement of the local findings, the patient was discharged in good general condition from the inpatient care on the 22^nd^ day of life. The subsequent dressing changes were performed in an outpatient setting. On the 24^th^ day of life, a black crust of 2×1.5 cm was removed at the lateral foot margin. Underneath, new epithelialized skin appeared. Fresh epithelialization appeared on the edge of the other wounds. From this point on, we decided to perform the dressings with dry, and sterile swabs. 

On the 34^th^ day of life, the last crust was removed from the small toe and the big toe. Below, rosy, well-perfused skin showed a capillary refill time of less than 2 seconds. The foot was now healed without any open spots or necrosis. The skin on the leg was only slightly dry and flaky. Like the rest of the body, the foot could now be treated with baby oil. The check-up intervals had been extended and were carried out on a monthly basis. On the 95^th^ day of life, the wounds were completely healed. 

Despite our efforts, the small toe showed flexion contracture compared to the right foot. The soft tissue of the small toe and its toe nail were laterally misrotated, the bone underneath, however, was palpable in an axis appropriate position (Figure 1-3). One year after the incident, a correction of the post-burn scar contracture was discussed with the parents. It was agreed to leave the nail bed in this situation for the time being and to carry out a correction at an advanced age.

We performed a Z-plasty along the contracture. The shortened flexion fold was incised and the toe was stretched in full extension. The defect size was measured in this position and at the lateral edge of the foot, outside of the instep area, a full thickness skin graft was removed and inserted into the defect. At the postoperative control, the child’s parents and we were satisfied with the aesthetic and functional outcome.

## DISCUSSION

To our knowledge, this is the first case report dealing with initial non-surgical combustion therapy in a newborn. The primary surgical approach has been reported elsewhere.^5^ The extent of the burn was about 1% of the body surface of the child. There was no life-threatening situation at any time. Estimation of burn depth is difficult and often appreciated by clinicians subjectively.^6^ Depth and gravity of the combustion can still evolve in the first 24-72 hours and a re-estimation of the area after some time is often necessary.^2^

The clinical care of burnt children requires a multidisciplinary therapy concept.^1^ According to recent studies, it is possible to treat up to 90% of burn patients on an outpatient basis. This approach is also applicable to pediatric patients.^7^^,^^8^ Long-term stays of burn patients bear the risk of infection with multidrug-resistant microorganisms and thus increase the overall risk of the patient. Yet, a stationary stay involves a more controlled therapeutic environment and thus allows a more effective immobilization to prevent dressing dislocations.^3^

Excessive burns of the foot or hand can damage tendons as well as joints and often require the use of microvascular or pedicled flaps. There are many innovative techniques in the surgical treatment of burns.^9^^,^^10^ Since the number of burn victims is high, especially in developing countries, standardized and cost-effective therapies should be made available.^11^^,^^12^ As surgical therapy bears the risk of possible mutilations and limitations of functionalities crucial for the development of the foot, we favored an initial non-surgical approach. Surgery for post-burn scar contractures should be performed at least one year after the burn injury and not during the active phase of wound healing and scarring.^13^

Modern wound therapy of burns is carried out according to the principle of less frequent dressing changes, this allows the burn wound to have a better re-epithelialization.^3^ We preferred fatty gauze dressing over the usual wound treatment with coated foam, as this allowed us to better cover the complicated surface structures of the child‘s foot and toes. Adaptic® consists of a network of cellulose acetate fibers coated with a petrolatum emulsion, contains surfactant and reduces the surface tension and thus allows easy passage of exudate.

We have decided to switch from Jelonet^TM^ to Adaptic®, as it is easier to remove and significantly less pain is associated with the removal of Adaptic®. Adaptic® requires less soaking and causes less maceration than Jelonet^TM^.^14^ After removal of the necrosis and visualization of the underlying juvenile keratinocytes, we initiated a switch from Adaptic® to dry, sterile swabs. It is believed that direct contact with fatty wound dressings may adversely affect cell growth and survival of keratinocytes in the early wound healing phase. 

Keratinocytes that were in contact with Adaptic® dressing showed increased mortality, a decreased division rate, and changes in cell morphology, increased LDH liberation, and increased cell damage in an in vitro study.^15^ One of the basic principles of wound treatment is to create optimal conditions and allow the human body to regenerate. There is evidence that newborns have a much higher potential for wound healing than adults. Umbilical cord blood (UCB) and thus also the blood of the newborn is rich in stem cells such as mesenchymal stem cells (MSCs), hematopoietic stem cells (HSCs), endothelial progenitor cells (EPCs) and very small embryonic-like stem cells (VSELs). They could play a role in tissue repair after birth or postpartum injuries in children.^16^

Stem cells are immature progenitor cells that are capable of self-renewal of various tissues.^17^ MSCs are involved in the regeneration of tissue injuries and are characterized by a high proliferation rate.^18^ MSCs are the most commonly used stem cells. They secrete cytokines and numerous growth factors, such as epidermal growth factor (EGF) and fibroblast growth factor (FGF), which both play an integral role in skin rejuvenation and wound healing. This is accomplished by collagen synthesis of human dermal fibroblasts (HDFs).^19^

Umbilical cord blood derived mesenchymal stem cells (UCB-MSCs) express higher amounts of wound healing factors than other MSCs. They cause migration, proliferation and collagen synthesis in fibroblasts. In addition, they support wound closure and re-epithelialization.^20^^,^^21^ The presence of these cells in the neonatal blood circulation results from hypoxia and the rise of cytokines induced by multiple small tissue and organ injuries during birth. This mobilization of stem cells into the blood of the newborn is regarded as a type of autologous physiological stem cell therapy.^18^

It is assumed that embryotic stem cells survive into the adult age and play an important role as a backup population. The presence of pluripotent stem cells in adult tissues has already been demonstrated. During organ and tissue damage, it was observed that the concentration of very small pluripotent embryotic stem cells in the peripheral blood increases.^20^ It was demonstrated that pluripotent stem cells are mobilized in the peripheral blood as response to tissue damage, as well as to burns.^21^

The use of mesenchymal stem cells for the therapy of radiation induced burns has already been described.^22^ Positive effects of mesenchymal stem cells on the regeneration of severe burns had been confirmed in an animal experiment. MSCs migrate via the bloodstream to the damaged tissues. This phenomenon, described as homing, is not yet well understood. Blood flow is the key to wound healing and therefore, neovascularization by Vascular endothelial growth factor (VEGF) and basic fibroblast growth factor (FGF2) is of great importance.^18^

The use of mesenchymal stem cells from umbilical cord blood for scarless wound healing is a topic of great interest. These stem cells express the surface markers CD44, 73, 90 and 105. The antifibrotic wound healing factor HGF, which is involved in scarless wound healing, is particularly expressed in UCB-MSCs. Accelerated wound healing by implantation of UCB MSCs has not been demonstrated yet. These gene expression profiles indicate that UCB-MSCs could be a stem cell source for unscarred wound healing. It can be assumed that neonatal blood has the same potential.^23^

The skin is the organ with the highest number of stem cells and therefore capable of extraordinary regeneration after injuries or burns.^23^ Apart from MSCs other stem cells, such as epithelial stem cells, adipose-derived stem cells and fibroblasts, might play important roles in tissue regeneration.^24^ We assume that apart from the primary debridement, it was mainly the conservative approach under sterile conditions, the antibiotic prophylaxis to prevent bacterial infections and the increasingly well-understood effect of stem cells in the blood of newborns that finally led to this satisfactory result. The functional result, however, could only be improved by surgical intervention. It showed that in case of long-term immobilization of toes, more attention should be paid to the proper position to prevent scar contracture and deformity.
